# A Case Study and State of Science Review: Private versus Public Healthcare Financing

**DOI:** 10.5539/gjhs.v4n1p118

**Published:** 2012-01-01

**Authors:** Poongodi Sampath, Donna M. Wilson

**Affiliations:** Faculty of Nursing, University of Alberta Edmonton, Alberta, T6G 1C9, Canada Tel: 1-780-437-1929 E-mail: sampath@ualberta.ca; Faculty of Nursing, University of Alberta Edmonton, Alberta, T6G 1C9, Canada Tel: 1-780-492-5574 E-mail: donna.wilson@ualberta.ca

**Keywords:** Case study, Systematic review, Healthcare-insurance, Wait times, Public policy, Healthcare equity, Universality, Privatization

## Abstract

Medicare is a popular program in Canada that offers universal access to medically-necessary healthcare services for all Canadians through a public insurance plan in each province. In spite of its popularity, healthcare privatization has been debated, often over concerns about wait times for select healthcare services. A case report focused on the 2005 Supreme Court’s response to the “*Chaoulli v. Quebec*” challenge of the Quebec law banning the purchase of private health insurance for publicly-insured services is presented, along with findings from a state of science review to determine if there would be any benefit from adopting the United States model of private health insurance. This review reveals private health insurance would have significant negative implications, especially by creating inequity in healthcare access for low-income groups. Further study is needed to determine whether Canada’s publicly-funded healthcare system would benefit in any way from increased private financing.

## 1. Introduction

Medicare, a source of pride and national identity for many Canadians, is a prominent social program that has offered public insurance coverage for all Canadian citizens since 1966 to ensure universal access to medically-necessary healthcare services (Thornhill, Law, Clements, & Stipich, 2008; White & Nanan, 2009; Yalnizyan, 2006). In spite of the widespread popularity of Medicare, healthcare privatization has been a contentious recurrent issue (Brunet, 2011; Schraeder, 2006). Private insurance for publicly-insured healthcare services has been proposed as a solution to ameliorate wait times for select healthcare services in what is now an entirely public healthcare system (Skinner, 2009). Canada’s healthcare policy approach of prohibiting competitive private insurance for publicly-funded healthcare services has been noted as “extreme” by proponents of healthcare privatization; particularly as most other developed countries have some form of mixed public and private health insurance with varying levels of government regulation (Skinner, 2009). A case report on the 2005 Supreme Court’s response to the “*Chaoulli v. Quebec”* challenge of the law banning the purchase of private health insurance for publicly-insured services in the Canadian province of Quebec is presented below, following an outline of the value of case study research for informing policymaking. Following this, findings are presented from a state of science review that was undertaken in 2011 to determine if there would be any benefit from adopting what Canadians perceive to be the “United States’ model” of private healthcare insurance. The privatization issue discussed in this paper is not healthcare delivery (i.e. whether a public or private organization delivers healthcare services) but instead healthcare financing (i.e. by public or private sources, or a combination of both). Public funding is a key component of the Canadian Medicare system, and if this foundation changes, so too could the nature of Medicare.

## 2. The Value of Case Studies for Informing Health Policy and Policy Making

A case study involves an in-depth analysis of a specific phenomenon in its real-life context (Gagnon, 2010). Case study research is encouraged, as this type of inquiry can focus on single or multiple cases to produce or challenge a theory; and/or explain, explore, or describe a complex real-life situation or quandary (Gerring, 2007; Yin, 2003). Case studies in health services research are particularly valuable as they enable a better understanding of a large topic by focusing on key components (Gerring).

The typical case study method has five steps. The first is to determine whether the case study is the appropriate method to answer the research question (Gagnon, 2010). A case study is appropriate if the purpose of the research project is to answer *how* and *why* questions about a particular case in its’ real-life context (Yin, 2009). The second step is to develop a research design, so as to collect the right types of data and have appropriate data analysis strategies (Yin, 2010). The next three steps involve data collection, analysis and interpretation of data, and dissemination of the findings (Gagnon, 2010; Yin, 1999). Data collection should be done in a structured manner, with the evidence collected from multiple sources triangulated to ensure reliability and validity. During the analysis and interpretation of data, the research team should identify possible explanations, such as *why* a particular policy is effective or ineffective. Based on the disseminated case study results, other jurisdictions may introduce the same policy, a variant of the policy, or avoid the policy. Thus, case studies enable policymakers to learn from mistakes and successes, and ultimately make better policy decisions.

### 2.1 A Case Report on Private Insurance for Medicare

As indicated above, one current major aspect of the healthcare privatization debate in Canada is the ban on private health insurance for medically-necessary healthcare services (Flood & Haugan, 2010). A case study on private health insurance in a province where this ban has been lifted should inform whether or not this policy has been successful in reducing wait times (Merriam, 2009). Private health insurance in Canada has been traditionally limited to services not covered under the public health insurance plan of each province, as expected through the 1984 *Canada Health Act* (Hurley & Guindon, 2008). The purchase of a private insurance for some publicly-insured services, often referred to as duplicate private health insurance, has also been specifically prohibited in six Canadian provinces, including Quebec (Flood, 2006).

The 2005 Supreme Court “*Chaoulli v. Quebec”* case was initiated by Dr. Jacques Chaoulli, a Quebec physician (originally from France), who was frustrated in his ability to practice privately due to governmental limits; and so challenged the law prohibiting the purchase of duplicate private health insurance in Quebec (Flood & Xavier, 2008; Monahan, 2006; Madore, 2006). Dr. Chaoulli had earlier come into conflict with the Quebec Health Insurance Board over his application to operate a private hospital (by opting out of the provincial health insurance plan), with this application refused by the Board (Tiedemann, 2005). The case involved Dr. Chaoulli’s patient, a Quebec resident, Mr. George Zeliotis, who had to wait one year for hip replacement surgery. Mr. Zeliotis’ attempts to purchase private health insurance to get earlier hip surgery failed, and they blamed the law prohibiting duplicate private insurance for his treatment delay (Tiedemann, 2005). However, some argued at the time that Mr. Zeliotis, who was 73 years-old with hip and heart problems, would not have qualified for private insurance even if it were available (Flood & Lewis, 2005). The Supreme Court ultimately ruled in favor of Dr. Chaoulli and Mr. Zeliotis, declaring that “Quebec laws preventing the purchase of private insurance, in the face of long wait lists for public treatment, violate guarantees within the Quebec Charter of Human Rights and Freedoms” (Flood & Xavier, 2008, p. 617). In a slim majority (four judges in favor and three against), Chief Justice Deschamps dismissed the Quebec government’s claim that prohibiting duplicate private health insurance was necessary to protect the quality of the province’s publicly-funded healthcare system (Flood, 2006). This Supreme Court’s decision only applied to the *Quebec Charter and* not the *Canadian Charter of Rights and Freedoms*; hence the laws preventing the purchase of duplicate private health insurance remained legitimate in other provinces, such as Alberta and Ontario (Flood & Xavier, 2008).

As a consequence of the “*Chaoulli v. Quebec”* decision, the Quebec National Assembly passed Bill 33 in December 2006 to lift the ban on private health insurance for three publicly-insured surgical procedures - total hip replacement, knee replacement, and cataract removal (Flood & Xavier, 2008; Mehra, 2008). By then, Services Quebec (2011a) had already established a wait time guarantee in 2005 such that if a patient had to wait for more than six months for any of the three surgeries then the government would pay for their treatment in a private clinic in that province or elsewhere. This wait time guarantee was designed to reduce equity concerns as not all Quebec residents had the financial means to pay for private healthcare insurance and thus benefit from earlier surgery. However, as the specified treatments, when delivered in private clinics, were publicly-funded; this guarantee made Bill 33 redundant (Flood & Haugan, 2010). Since then, due to low public demand, the market for duplicate private health insurance did not grow in Quebec or anywhere else in Canada (Hurley & Guindon, 2008). However, the market for duplicate private health insurance could still grow in Quebec if this wait time guarantee does not operate properly (Flood & Haugan, 2010). According to Services Quebec (2011b), 55% of current patients requiring hip replacement surgeries receive their treatment within three months, while 85% of these patients receive treatment within six months. The Canadian Institute for Health Information (2010) similarly found the wait times for hip replacement surgeries in Quebec between the years 2006 to 2009 showed no evidence of change, with 88% of patients treated within six months over this time period. Although a six month guarantee is present for all residents of Quebec, individual wait times are not rigidly fixed anywhere in Canada, as patients are prioritized on the basis of severity of symptoms and not the length of their waits (Gaudet *et al.*, 2007; Services Quebec, 2011c). The Canadian healthcare system is designed to operate on a triage system, where the most ill persons are treated first; and with this prioritization contributing to cost-effectiveness and maximal health services utilization.

## 3. State of Science Review

As part of the “*Chaoulli v. Quebec”* case study, a state of science review was conducted to examine whether the Canadian healthcare system would benefit from adopting the United States’ model of allowing more private financing options. The United States was chosen for comparison over other countries because it is similar to Canada in many ways; however, it differs markedly with respect to healthcare insurance and healthcare access (Siddiqi, Zuberi, & Nguyen, 2009). Private healthcare insurance is dominant in the United States.

**Figure 1:**
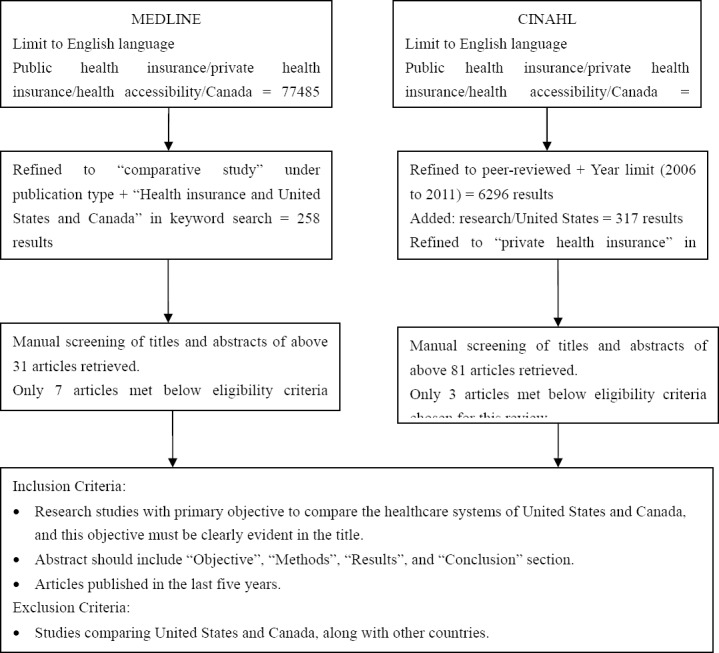
Methodological steps in systematic review

State of science or systematic reviews are an efficient method of identifying and reviewing literature in a highly structured manner, in order to provide an evidence base for a practice or policy (Whiting, 2009). The electronic library databases searched were MEDLINE and CINAHL, as these are the two most commonly used health library databases (see Figure 1). The keywords used in the search were: private health insurance, public health insurance, healthcare accessibility, Canada, and/or United States. The search was limited to English-language literature and further limited to research articles published in the last five years, as evidence from older studies was thought to be less relevant to the current context for informing health policy decisions. This search found several articles have been published using data from the 2002-2003 Joint Canada/United States Survey of Health (JCUSH). Of the 10 studies that initially met all eligibility criteria for review, four were excluded because they were from the same data source (i.e. JCUSH) and because these studies focused on different issues (e.g., access to prescription drugs, access to primary and preventative care services, etc.). A study by Blackwell, Martinez, Gentleman, Sanmartin, and Berthelot (2009), that used the “JCUSH” data, was retained as it took into account several measures of socio-economic status, was inclusive of two of the other excluded studies; and was focused on the topic of interest for this case study. In total, six articles (with one of these a systematic review) were reviewed, with the information gained from each summarized in Table 1.

**Table 1:** Results of studies reviewed to compare the performance of United States and Canadian healthcare systems, with focus on health insurance and socio-economic status

Reference	Aim of Study	Methods	Findings	Reviewer’s Comments
Blackwell, Martinez, Gentleman, Sanmartin, & Berthelot, (2009)	To examine the factors associated with the utilization of physician and hospital services among adults in Canada and the United States (US), with a focus on socio-economic status (SES) and healthcare insurance coverage.	Study used data from the 2002-2003 “Joint Canada/United States Survey of Health”. Country-specific multivariate logistic regressions were conducted to predict healthcare utilization after controlling for predisposing factors, enabling resources (e.g., health insurance), and perceived need for healthcare.	Adults in Canada and the US exhibited similar patterns of hospital utilization, and SES (including health insurance coverage) played no explanatory role. Instead, only the individual’s predisposing characteristics (e.g. age and sex) and his/her need for healthcare predicted utilization of hospital services in both Canada and the US.	This study was reviewed as it could explain whether the universal access to hospital services in Canada affects the rate of utilization, when compared to that of the US where private health insurance play a role in access to these services. Since, no difference in service utilization based on the type of insurance coverage was found, this study is determined as offering mixed results.
Gorey, Luginaah, Holowaty, Fung, & Hamm, (2009)	To determine whether SES has a differential effect on waits for surgical and adjuvant radiation treatment (RT) of breast cancer in Canada and the US.	Data was obtained from Ontario and California cancer registries between 1998 and 2000. Residence-based SES data were taken from censuses. Median waits were compared within and between countries using Mann-Whitney U-test.	There were significant associations between lower SES and longer surgical waits and lower access to adjuvant RT waits across diverse places in California. None were observed in Ontario. However, relatively high-income women with breast cancer in Ontario typically waited one to two months longer for adjuvant RT than their counterparts in California did.	This study was reviewed as it could explain whether the US system had shorter waits to cancer care than that of Canada. High-income US patients had shorter waits than Canadians, but since greater inequity was found in the US system, the study is determined as offering mixed results (shorter wait times for high-income groups in the US versus greater equity in Canada).
Guyatt *et al*., (2007)	To perform a systematic review of studies comparing health outcomes in the US and Canada among patients treated for similar underlying medical conditions.	Research of multiple bibliographic databases and resources. Study results were masked before determining study eligibility. For all eligible studies, original authors were asked for additional specific information and also to confirm accuracy of the information drawn from their study.	Of the 38 studies that met the study’s eligibility criteria, 14 favored Canada, 5 favored US, and 19 showed mixed results. The only condition in which results consistently favored one country was end-stage renal disease, in which Canadian patients fared better. Overall, the authors concluded that patients cared for in Canada have superior health outcomes than the US.	This study was reviewed as it could identify whether the US system, with a large private health insurance sector, is able to achieve better health outcomes than that of the Canadian system. Since the Canadian system was found to be cost-effective, while achieving equal or better health outcomes than that of the US, this study is noted as favoring Canada.
Krajewski, Hameed, Smink, & Rogers, (2009)	To determine whether or not there is a difference in access to emergency operative care between Canada and the United States based on socio-economic status (SES), given the difference in health insurance coverage among these two countries.	Data obtained from Canadian Institute for Health Information database and the US Nationwide Inpatient Sample, and included all patients diagnosed with acute appendicitis from 2001 to 2005. Univariate and multivariate analyses were performed to determine the odds of appendiceal perforation at different levels of SES in each country.	In Canada, there was no difference in the odds of perforation between income levels. In the US, there was a significant, inverse relationship between income level and the odds of perforation. The authors conclude this difference in access to emergency operative care could result from concern over the ability to pay medical bills or the lack of a stable relationship with a primary care provider that can occur outside of a universal healthcare system.	Treatment delays in the case of appendicitis would increase the risk of perforation. Since the study found the risk of perforation increased with each decreasing income level in the US patients but no such difference existed in Canada, it is evident that the Canadian system is successful in ensuring equitable access to emergency operative care, without financial barriers. Thus, this study is determined as favoring the Canadian healthcare system.
Li, Lau, McCarthy, Schull, Vermeulen, & Kelen, (2007)	To compare emergency department (ED) visit rate in the US and Ontario, Canada, according to demographic and clinical characteristics.	A cross sectional study with a sample of 40,253 ED visits included in the National Hospital Ambulatory Medical Care Survey in the US, and National Ambulatory Care Reporting System in Ontario, Canada.	The study found annual ED visit rate in the US was identical to the rate in Ontario, Canada; and concluded that differences in health insurance coverage may not have a substantial impact on the overall utilization of emergency care.	With no link found to the type of insurance coverage and overall utilization of emergency care, the study’s authors ponder that other factors may be contributing to the ED overcrowding in both countries. This study thus provided mixed results.
Rowe, Bota, Clark, & Camargo, (2007).	To compare emergency department (ED) asthma management and outcomes between Canada and the US, since acute asthma is the most common ED presentation in both countries.	A prospective cohort study of 69 American and eight Canadian EDs was conducted. Patients aged two to 54 years who presented with acute asthma underwent a structured ED interview and telephone follow-up two weeks later.	In terms of asthma chronicity and presentation to the ED, the US patients more often reported barriers to access primary care, demonstrated poor asthma control, and presented with suboptimal preventive medical management than their Canadian counterparts.	This study was reviewed as it could identify whether the universal access to primary care services in Canada play a role in health outcomes related to asthma. The study did find poor asthma control in the US patients without health insurance, and thus the study’s results favored Canada.

### 3.1 Health Insurance Evidence: Private versus Public

The evidence gathered through this literature review revealed, as outlined in Table 1, that encouraging private health insurance for medically-necessary healthcare services would create socio-economic equity concerns. Hence, its’ adoption as a means to reduce wait times in the public system is not recommended. This conclusion is based on finding that three studies favored Canada’s public healthcare insurance (two through evidence of better health outcomes, and one showing better public access to primary care services) and three provided mixed results. None clearly favored the United States’ private healthcare insurance or healthcare system in terms of equity, efficiency, or other factors (see Table 2). Guyatt *et al*.’s (2007) systematic review of studies that compared health outcomes in Canada and United States concluded that “Canada’s single-payer system, which relies on not-for-profit delivery, achieves health outcomes that are at least equal to those in the United States at two-thirds the cost” (p. E36). Of the 38 studies reviewed by Guyatt *et al*. (2007), 10 studies included extensive statistical adjustment and among which five favored Canada, two favored the United States, and three showed mixed results. Although neither Canada nor the United States had consistent superior healthcare outcomes, the outcomes for Canada were more often superior to those of the United States for patients with similar underlying medical conditions (Guyatt *et al.*, 2007). For instance, Canadian outcomes appeared superior in head and neck cancer, and possibly for various types of cancers for low-income groups; while breast cancer survival rates were better in American women when compared to Canadian women. The findings from the five other reviewed studies follow to better illustrate the identified evidence and conclusion reached.

**Table 2:** Summary of findings

Results favored Canada	3
Results favored United States	0
Mixed or equivocal results	3

Gorey, Luginaah, Holowaty, Fung, and Hamm’s (2009) study found high-income breast cancer patients in the United States with private health insurance had shorter wait times for surgery and radiation treatment than their Canadian counterparts. However, the same study found remarkable equity in Canadian breast cancer care, in contrast to a stark socio-economic inequity in access to such care in the United States. A study by Li, Lau, McCarthy, Schull, Vermeulen, and Kelen (2007) used a nationally representative sample of 40,253 emergency department (ED) visits to compare the ED visit rate in the United States and Ontario, Canada. They found that the annual ED visit rates in the United States (39.9 visits per 100 population) was almost identical to the rate in Ontario, Canada (39.7 visits per 100 population); and hence concluded that differences in “health insurance coverage may not have a substantial impact on the overall utilization of emergency care” (Li *et al.*, p. 582). Similarly, a study by Blackwell *et al*. (2009) examined whether socio-economic status (SES) and healthcare insurance coverage were associated with difference in the utilization of hospital services among adult patients in Canada and the United States. While the study found no difference in hospitalization based on SES, Blackwell *et al*. noted this lack of difference could be due to hospitalizations resulting from emergency situations in many cases and hence these are likely to occur irrespective of the patient’s health insurance coverage status. This argument is supported by their finding that adults who lacked insurance coverage in the United States stayed fewer nights in the hospital when compared to insured Americans. Krajewski, Hameed, Smink, and Rogers (2009) used information on patients diagnosed with acute appendicitis from 2001 to 2005 to determine whether Canada and United States differed in terms of access to emergency operative care. They found no difference in the odds of appendiceal perforation at different levels of SES in Canada; however, there was a significant inverse relationship between the odds of appendiceal perforation and income levels in the United States. Based on their results, Krajewski *et al*. (2009) concluded that unlike Canada, the ability to pay and/or the patient’s SES determines access to emergency operative care in the United States. In another comparative study, Rowe, Bota, Clark, and Camargo (2007) examined differences in acute asthma presentations to hospital emergency departments across Canada and the United States. As asthma is a chronic disease, patients in the United States more often reported access barriers and they were less likely to be insured (Rowe *et al.*, 2007). In short, this state of science review included evidence from six articles, and these clearly showed no immediate or other benefit from private healthcare insurance.

### 3.2 State of Science Literature Review Discussion

The findings from this literature review suggest that instead of Canada allowing private healthcare insurance or financing measures, the healthcare system in the United States should consider moving to a publicly funded health care insurance system. The rationale for this is that the United States has a major wait time problem of its own (Gorey *et al.*, 2009). The wait times in a multi-payer system like that of the United States are much less transparent than the wait times in Canada’s single-payer system. For instance, among the 47-50 million people without any healthcare insurance in the United States, many cannot afford to be on any waiting lists (Gorey *et al.*, 2009). While no concrete evidence was found to prove that the Canadian healthcare system would react adversely from adopting the United States model, experimenting with private healthcare financing options would be dangerous since Canada’s trade treaties make it difficult to reverse commercialization reforms once initiated (Bryant, 2009). The case study of the “*Chaoulli v. Quebec”* challenge in Quebec is another illustration of the lack of value and need for private healthcare insurance in Canada.

## 4. Conclusion

This case report of a controversial policy permitting private health insurance in the Canadian province of Quebec hopefully convinces readers of the significance of conducting case studies for informing policy. Case studies are robust, with many sources of information of possible interest and relevance to enhanced policy-making. The evidence from a state of science review presented in this paper also clearly suggests the Canadian healthcare system would not benefit from adopting the United States’ healthcare funding model, where private health insurance dominates. Instead, it indicates that if Canadian policymakers decide to adopt private financing options as a quick fix for wait times in the public system, this apparent remedy would have long-term negative implications for Canada, especially by creating inequity in healthcare access. Low-income groups would suffer as they are less able to purchase private insurance. As this “*Chaoulli v. Quebec”* case study illustrates, Canadians so far have upheld healthcare equity through public funding instead of embracing private healthcare financing options.
